# A comparative study of sexual dysfunction involving risperidone, quetiapine, and olanzapine

**DOI:** 10.4103/0019-5545.58291

**Published:** 2009

**Authors:** Anil Kumar M. Nagaraj, Nagesh B. Pai, Satheesh Rao

**Affiliations:** Department of Psychiatry, Mysore Medical College & Research Institute, Mysore- 570001, Karnataka, India; 1 Department of Graduate School of Medicine, University of Wollongong, Australia, India; 2 Department of Psychiatry, K S Hegde Medical Academy, Deralakatte, Mangalore 575018, Karnataka, India

**Keywords:** Olanzapine, quetiapine, risperidone, schizophrenia, sexual dysfunction

## Abstract

**Background:**

With the advent of newer antipsychotic drugs, side effects such as sexual dysfunction have been a major contributor toward treatment compliance. There are only a few studies that have compared different atypical antipsychotic agents regarding sexual dysfunction. We have not come across any data in this area on Indian population.

**Aims:**

To determine and compare the frequency of sexual dysfunction associated with risperidone, olanzapine, and quetiapine, among patients with clinically stable schizophrenia.

**Settings and Design:**

It is a cross-sectional hospital-based study. The subjects were recruited for the study by the purposive sampling technique.

**Materials and Methods:**

The total sample size was 102, consisting of 25 each in the quetiapine and risperidone groups, 22 in the olanzapine group, and 30 healthy volunteers. A Brief Psychiatric Rating Scale and Sexual Functioning Questionnaire (SFQ) were administered. The Kruskal Wallis test was used to compare the variables in the demographic data and the mean chlorpromazine equivalent doses of the study groups. To analyze the sexual dysfunction, the mean scores on all the domains of sexual functioning in SFQ were compared across the study groups using the Chi square test, for proportions.

**Results and Conclusion:**

Twenty-three percent of the healthy volunteers had some impairment in one or more domains of sexual functioning. For the medication groups this was 96, 88, and 90%, respectively for risperidone, quetiapine, and olanzapine. However, there was statistically no significant difference across the study groups although it was relatively less with quetiapine.

## INTRODUCTION

Sexual function is the physiological capacity to experience desire, arousal, and orgasm. Sexual dysfunction can result from a wide variety of psychological and physical causes. Among drugs, antihypertensives, diuretics, antihistamines, antidepressants, benzodiazepines, and antipsychotics are the common agents associated with sexual dysfunction.[1] Schizophrenic patients can develop sexual dysfunction that may not be related to drugs. Studies have shown that a majority of untreated schizophrenic patients have a reduced desire for sex, more in females as compared to males, although arousal and ejaculatory functions remain relatively intact. The schizophrenic men often limit their sexual activity to masturbation, as the negative symptoms limit their ability to maintain relationships.[2] However, while on treatment, they may experience erectile dysfunction and orgasmic difficulties as adverse effects of the medicines, that is, antipsychotics, unless they have no primary organic pathology or comorbid medical conditions contributing to the sexual dysfunction.[3,4] Thus, the major impact on sexual functioning in schizophrenic patients is by antipsychotics. There are sufficient studies that have looked into sexual dysfunction due to typical antipsychotics as well as studies that have compared typical and atypical antipsychotic agents.[5–9] However, there are only a few studies that have compared different atypical antipsychotic agents for sexual dysfunction.[10,11] The authors have not come across such studies among the Indian population.

## MATERIALS AND METHODS

The study sample was taken from the Psychiatry Outpatient Department and it consisted of 72 patients with clinically stable schizophrenia meeting the ICD-10 criteria; as well as 30 healthy volunteers from among the staff of the hospital and caregivers of patients who were willing to participate in the study. This is a cross-sectional, hospital-based study. After obtaining the local ethical committee clearance, the subjects were recruited for the study by the purposive sampling technique during August 2005-April 2006. The sample (N = 102) was divided into four groups [Table 1]. Group one (G_1_) consisted of 25 patients on risperidone, group two (G_2_) had 25 patients on quetiapine, group three (G_3_) was made up of 22 patients on olanzapine, and group four (G_4_) had 30 healthy volunteers. These drugs were not administered for the purpose of the study. The patients, who were maintaining remission on one of these drugs, taken in the oral form tablets, were enrolled into the study during their regular follow up, after their written consent. Study-related assessments were done on the same day of selecting the patients for the study.

**Table 1:** Subtype of schizophrenia across the three drug groups

Study groups	Paranoid	Hebephrenic	Catatonic	undifferentiated	Unspecified
G_1_	14	2	1	7	1
G_2_	15	1	0	9	0
G_3_	16	0	0	6	0

G_1_ = Risperidone group; G_2_ = Quetiapine group; G_3_ = Olanzapine group

The sample consisted of male patients between 18-50 years of age, sexually active (not abstinent) and on regular treatment with a stable dose of risperidone, quetiapine, or olanzapine for at least six weeks after achieving clinical stability. Female patients were not included in the study as the types of questions in the SFQ were not suitable for the conservative female population of this locality or for their cooperation to answer them. Remission was defined by a score of less than 4 on all items of BPRS.[12] Patients having other comorbid medical and psychiatric illnesses as well as primary sexual dysfunction were not included. Furthermore, those on more than one antipsychotic drug or other drugs affecting sexual function, like benzodiazepines, antidepressants, and antihypertensives were also not included. The only allowed medication along with the above-mentioned antipsychotics was trihexyphenidyl, given to control extrapyramidal side effects.

The sociodemographic and clinical information sheet, BPRS, and SFQ[6] were the tools used for assessing the patients. The SFQ was the modified version of a questionnaire used by Burke *et al*.[13] For those who did not know English, a vernacular translation was administered by the author. The SFQ asked detailed questions about the physical aspects of sexual functioning including libido, physical arousal, masturbation, orgasm (including painful orgasm), and ejaculation. It had been further modified so that it had subscales for the different areas of sexual functioning. It was not necessary for the subject to have a partner in order to complete it. The scale, though not tested adequately for validity, had good reliability: Cronbach's α = 0.90; Guttman's split-half reliability = 0.86. For the purpose of statistical analysis, an arbitrary cut off point of one standard deviation above the mean was taken as the threshold above which sexual dysfunction was said to be present. Taking that into consideration, the subscales of the questionnaire served as continuous variables, which were studied across the study groups.

Patients with clinically stable schizophrenia, as per ICD-10 criteria, attending the Department of Psychiatry were interviewed after taking informed consent. After collecting the required sociodemographic and clinical data from the patients, they were rated on BPRS, to rule out any active psychopathology. Subsequently, they were rated on the SFQ to determine the dysfunction in the phases of desire, arousal, and orgasm. Healthy volunteers who were medically fit and not on any medication were asked to fill a sociodemographic data sheet as well as sexual functioning questionnaire. This data from healthy volunteers was collected for statistical purposes, to set a normal mean score on SFQ.

### Statistical analysis

The statistical analysis of data was performed using SPSS for Windows (version 12.0) and Microsoft Excel.

Descriptive statistics were applied to obtain the means and frequencies of sociodemographic and clinical data of the sample. Subsequently, the Kolmogorov-Smirnov test was used to check for normality of distribution of data on the sexual functioning questionnaire (SFQ). It was found that most of the data was not normally distributed. Thus non-parametric tests were used for comparative statistics. The Kruskal Wallis test was used to compare the continuous variables in demographic data as well as mean chlorpromazine equivalent doses of the three study groups.

To analyze the sexual dysfunction, the mean scores of the sexual functioning questionnaire on the domains of desire, arousal/erection, orgasm/ejaculation, and overall sexual impairment was obtained. The SFQ is designed such that the higher the score, more severe is the sexual dysfunction. An arbitrary cut off point of 1 SD above the mean score of healthy volunteers (G_4_) was taken as the threshold above which sexual dysfunction was said to be present. The mean scores on all the domains were compared across the study groups using the Chi square test, as proportions and level of significance were calculated from this.

## RESULTS

The groups were evenly matched with respect to key clinical variables, such as, age, duration of illness, duration of clinical stability, and treatment duration as shown in Tables 2 and 3. A majority of them were educated above higher secondary school. Illiterates constituted 7.8%. The occupation of most of the study subjects was agricultural farming (26.5%) and the family income of most of them (28.4%) was in the range of Rs 2000-3000 per month. Two-thirds of the subjects were from extended families and the same proportion was married. A majority of them had received the diagnosis of paranoid schizophrenia (62.5%). Out of the 72 patients, only six were clinically stable beyond one year (four in the risperidone group and two in the quetiapine group). The rest of them were stable for less than one year, but more than six months at the time of data collection. Though clinically insignificant, the risperidone group had comparatively a lesser duration of active illness and a greater duration of clinical stability. Further, the duration of medication use at the time of assessment was also less, although not clinically significant, for the risperidone group.

**Table 2:** The continuous demographic and clinical variables across the four groups

Variables	Mean ± SD			
	
	G_1_ (n = 25)	G_2_ (n = 25)	G_3_ (n = 22)	G_4_ (n = 30)
Age (yrs)	28.58 ± 7.60	28.76 ± 6.39	30.68 ± 9.65	30.23 ± 7.91
Years of education	8.64 ± 4.39	9.80 ± 4.66	8.81 ± 4.58	12.13 ± 4.47
Duration of illness (months)	66.50 ± 58.46	80.72 ± 75.72	70.13 ± 51.14	NA
Clinically stable (months)	10.12 ± 11.05	8.44 ± 16.03	5.64 ± 2.85	NA
Duration of treatment (months)	6.83 ± 5.87	6.13 ± 3.25	6.42 ± 3.79	NA

NA = Not applicable; G_1_ = Risperidone group; G_2_ = Quetiapine group; G_3_ = Olanzapine group; G_4_ = Healthy volunteers

**Table 3:** The comparison of sociodemographic and clinical variables across the three medication groups

Variables	Mean ranks			*P*
		
	G_1_ (n = 25)	G_2_(n = 25)	G_3_(n = 22)	
Age	34.98	36.06	38.73	0.822
Years of education	34.28	39.62	35.48	0.636
Occupation	33.46	38.08	38.16	0.650
Income	38.22	36.92	34.07	0.777
Family type	34.60	38.92	35.91	0.664
Mother tongue	34.10	35.74	40.09	0.579
Marriage	36.48	37.92	34.91	0.831
Domicile	38.10	30.60	41.39	0.100
Religion	37.94	34.42	37.23	0.753
Duration of illness	34.62	37.78	37.18	0.852
Duration of clinical stability	40.28	33.72	35.36	0.515
Duration of treatment	34.78	37.04	37.04	0.871

Kruskal Wallis test; *P* = NS

It was important to look for the normality of distribution of data on the SFQ, before applying for the statistical tests. This was analyzed by applying the Kolmogorov-Smirnov test. It was found that a majority of the variables were not normally distributed. The scores for desire in the risperidone and quetiapine groups and the overall sexual dysfunction scores for the risperidone, olanzapine, and healthy volunteer groups followed a normal distribution, but not the remaining majority of items. This is shown in Table 4. Thus nonparametric tests were applied to analyze the sexual dysfunction. As only clinically stable patients were selected, their scores on BPRS were not compared statistically.

**Table 4:** Test of normality on sexual functioning questionnaire scores

Items on scale	Study groups	*P*
Desire	G_1_ (N = 25)	0.156
	G_2_ (N = 25)	0.155
	G_3_ (N = 22)	0.075
	G_4_ (N = 30)	0.042
Arousal/Erection	G_1_ (N = 25)	0.000
	G_2_ (N = 25)	0.034
	G_3_ (N = 22)	0.034
	G_4_ (N = 30)	0.073
Orgasm/Ejaculation	G_1_ (N = 25)	0.000
	G_2_ (N = 25)	0.000
	G_3_ (N = 22)	0.000
	G_4_ (N = 30)	0.000
Overall sexual functioning	G_1_ (N = 25)	0.200
	G_2_ (N = 25)	0.010
	G_3_ (N = 22)	0.200
	G_4_ (N = 30)	0.200

Kolmogorov – Smirnov test

The mean (SD) daily doses of the three drugs were found to be 5.2 (1.65) mg, 468.0 (141.33) mg, and 13.86 (4.06) mg for risperidone, quetiapine, and olanzapine, respectively. Their mean (SD) chlorpromazine equivalent doses were 260.00 (82.60) mg for risperidone, 936.00 (282.00) mg for quetiapine, and 351.13 (101.61) mg for olanzapine. The chlorpromazine equivalent doses of the three drugs were compared using the Kruskal Wallis test, as the data here was also not normally distributed. This data is shared in Table 5. The quetiapine dose was significantly higher (*P* = 0.000) compared to the other drugs. These finding show that a higher chlorpromazine equivalent dose of quetiapine may be required compared to risperidone and olanzapine, for achieving and maintaining clinical stability in schizophrenia.

**Table 5:** Comparison of chlorpromazine equivalent doses of three study drugs

Drugs	Mean rank	Chi sq value	*P*
Risperidone	18.48	52.147	0.000
Quetiapine	59.92		
Olanzapine	30.36		

Kruskal Wallis test

### Sexual side effects — Frequency and severity

Sexual side effects across the three drug groups were compared on SFQ for frequency as well as severity of all the domains, that is, desire, arousal/erection, orgasm/ejaculation, and overall sexual impairment. The Sexual Functioning Questionnaire is a sensitive tool, with 38 items that assess sexual functioning. About 23% of the healthy volunteers had their score above 1 SD of the mean, thus having some impairment in one or the other domain of sexual functioning. For the medication groups this was 96, 88, and 90%, for risperidone, quetiapine and olanzapine, respectively. Desire was most commonly impaired in the risperidone group (80%) as compared to 72% in the quetiapine group, and 78% in the olanzapine group. Erectile dysfunction was most common in the olanzapine group (50%). It was 40% in the risperidone group and 36% in the quetiapine group. Orgasmic dysfunction was equally common to both the risperidone and quetiapine (32%) groups and 27% in the olanzapine group. This is shown in Table 6.

**Table 6:** Frequency of sexual dysfunction across the four study groups

Study group	Desire (%)	Arousal/erection (%)	Ejaculation/orgasm (%)
G_1_ (n = 25)	80	40	32
G_2_ (n = 25)	72	36	32
G_3_ (n = 22)	78	50	27
G_4_ (n = 30)	20	20	21

### Comparison of sexual dysfunction across study groups

The mean scores from SFQ on all domains of sexual functioning of the three medication groups were compared by the chi square test for proportions with the healthy volunteer group. Although the mean scores of the quetiapine group for overall sexual side effects, desire, and arousal/erectile impairment were less than the other two medication groups, there was no significant difference across the three groups in any of the domains of sexual dysfunction. The details of the mean scores and their comparison are given in Table 7.

**Table 7:** Mean scores of study groups on sexual functioning questionnaire and their comparison

Domains of sexual function	G_1_ (n = 25) Mean ± SD	G_2_ (n = 25) Mean ± SD	G_3_ (n = 22) Mean ± SD	G_4_ (n = 30) Mean ± SD	95% CI for difference in mean	*P* value
Overall sexual dysfunction	19.48 ± 3.65	17.44 ± 2.90	18.63 ± 3.94	9.86 ± 3.84	6.0 to 13.7	0.45
Impaired libido	4.84 ± 1.17	4.32 ± 1.24	4.50 ± 1.50	2.53 ± 1.38	1.1 to 3.9	0.79
Arousal/erectile dysfunction	5.04 ± 2.15	4.08 ± 1.11	5.22 ± 2.44	2.93 ± 1.76	1.1 to 4.6	0.61
Orgasmic/ejaculatory dysfunction	2.48 ± .29	2.52 ± 0.82	2.20 ± 0.78	1.46 ± 1.43	0.1 to 2.9	0.70

Chi-square test for proportions; *P* = NS

## DISCUSSION

### Overall sexual impairment

The diagnosis of sexual dysfunction is not absolute. Persons with adequate sexual functioning show enormous variability in frequency of sexual activity and desire; and normal sexuality may include an occasional dysfunctional moment. The literature addressing the western population report that about 10-15% of the normal population suffers from sexual dysfunction.[14] The SFQ recognized sexual dysfunction in 17% of the normal western population.[6] In the current study using the same SFQ, sexual dysfunction was recognized in 23% of the healthy volunteers. This is slightly higher than the western figures. However, the total mean score on SFQ of the healthy volunteers, in the original study, was 12.1 ± 6.9 and in the current study it is 9.86 ± 3.84. In other words, the severity of sexual dysfunction, (quantified by mean score) which has a greater impact, appears to be more or less the same.

In this study risperidone was associated with the most frequent overall sexual impairment (96%) compared to olanzapine (90%) and quetiapine (88%), although it was not statistically significant. Melkersson has also reported an overall sexual dysfunction of 89% due to risperidone.[15] Up to 93% of risperidone-treated patients reported an overall impairment of sexual functioning in yet another study.[16] Even in the case of olanzapine and quetiapine, a frequency of overall sexual impairment, comparable to our study, was observed in other studies.[10,16] Kelly and Conley had compared fluphenazine, risperidone, and quetiapine and reported a much higher degree of sexual dysfunction with quetiapine compared to all other previous studies. It was a randomized, double blind 12-week study, reporting overall sexual impairment as high as 50% with quetiapine as compared to 42% with risperidone and 78% with fluphenazine. The study also looked into the prolactin levels due to study drugs. An interesting aspect in this study was that quetiapine had overtaken risperidone in the frequency of sexual dysfunction, although risperidone caused the highest levels of prolactin elevation and quetiapine caused none. This indicated that prolactin elevation was not the only mechanism behind drug-induced sexual dysfunction, and that quetiapine could also cause a substantial degree of sexual dysfunction, although it was not associated with the elevation of prolactin levels.[16] Nonetheless, the present study supports this finding especially with respect to the higher frequency of quetiapine-associated sexual dysfunction.

Another recent study compared risperidone, olanzapine, and quetiapine for sexual dysfunction using a different questionnaire called Arizona Sexual Experience Scale (ASEX). It was also a cross-sectional study like the present one with a sample size of 238 (quetiapine-57, olanzapine-94, risperidone-87). The mean scores on ASEX were relatively low in the quetiapine group compared to the other two drugs, as in our study. The patients in all the treatment groups, nonetheless, experienced a moderately high degree of sexual dysfunction. However, the quetiapine group experienced a slightly lesser degree of sexual dysfunction, although it differed significantly only with the olanzapine group.[10] Our study supports the findings of this study too, with respect to the sexual side-effect frequency of quetiapine that is comparable to risperidone and olanzapine. This is contrary to the earlier evidence that assigned a better safety profile to quetiapine with respect to sexual functioning.[7,11]

### Desire

Impaired desire is the most frequently reported sexual dysfunction among all the medication groups in the present study. An assessment of changes in libido associated with psychotropic medications can be difficult, because psychiatric illnesses can significantly affect sexual interest. In symptomatic cases of schizophrenia with prominent negative symptoms, the frequency of sexual fantasy is much reduced and their sexual activity is reduced to masturbation.[17] The effects of antipsychotics on libido are not as well characterized as other forms of sexual dysfunction, in part because of the difficulty in measuring changes in libido. Nevertheless, several factors influence desire. Failure of erection may itself adversely affect a patient's desire. A patient's socioeconomic status and quality of life also influence his libido. Libido was the most frequently reported sexual dysfunction with both haloperidol (58%) and clozapine (50%), in one of the studies.[18] Of late, another study reported that impaired desire (44%) was the most common sexual dysfunction due to risperidone.[15] One more study reported that impaired libido is commonly seen even with quetiapine.[19] These finding are supported by the present study, which reports an impaired libido of 80% with risperidone, 72% with quetiapine, and 78% with olanzapine. However, considering the facts discussed earlier in this section, it would be difficult to conclude that the drugs are entirely responsible for the higher rate of impaired libido reported in this study. We cannot exclude the role of illness sharing to some extent the impairment of libido experienced by the patients. An elevated prolactin level is an important biological marker of impaired libido due to antipsychotics. This index can be of some help to know to what extent the drug and the illness are attributable for the impaired libido.

### Arousal/erection

Erectile problem was the second most frequent sexual side effect in the current study. Among the three drugs, olanzapine was most commonly associated with erectile dysfunction (50%) as compared to risperidone (40%) and quetiapine (36%). However, it was not statistically significant. While many studies reported desire to be the most common sexual side effect due to antipsychotics, a good number of studies inferred that erectile failure was also an equally common sexual side effect. However, it was easier to measure and quantify erectile dysfunction compared to libido, due to the availability of procedures like measuring nocturnal tumescence and penile plethysmography. Furthermore, it was also easier for the patient to appreciate and report his erectile problems compared to diminished libido. One study reported erectile difficulties associated with antipsychotic drugs in 38% schizophrenic patients,[19] followed by 47% in another study,[26] and 52% in yet another.[8] However, these studies included typical antipsychotics too. In one of the studies on Indian population, the erectile dysfunction was 53% with typical antipsychotics and 31% with atypical antipsychotics and it differed significantly (*P* = 0.025). However, the tool used for assessment was the UKU side effect rating scale. No comprehensive questionnaire was used.[9] The frequency of erectile dysfunction reported in the current study, however, falls in the range of that reported in earlier studies.

### Orgasm/ejaculation

In a majority of the published studies, orgasmic and ejaculatory problems were less commonly reported than the desire and erection problems associated with antipsychotics. This is especially true in the case of atypical antipsychotics. A common problem in assessing orgasmic and ejaculatory problems is the co-occurrence of erectile dysfunction. In such cases the patient cannot satisfactorily recognize their ejaculatory and orgasmic function. As he cannot achieve complete erection, he may not ejaculate and experience orgasmic joy even though his orgasmic capacity is intact. This limitation could not be answered in our study too. Orgasmic and ejaculatory problems were least affected among the patients in the present study. Thirty-two percent of the patients on risperidone and quetiapine and 27% of those on olanzapine had orgasmic/ejaculatory problems. In a study by Wirshing and co-workers, orgasmic and ejaculatory difficulties were found in 86% of the patients on risperidone as compared to 20% on clozapine. Nevertheless, the sample size was too small (n = 14 for risperidone and n = 5 for clozapine) and a Type II error was clearly evident.[20] One of the recent studies reported that patients on quetiapine had better orgasmic quality and ability compared to risperidone and fluphenazine.[16] However, in the present study the orgasmic capacity was equally impaired in both the risperidone and quetiapine groups. In the former study, the patients received drugs for only 12 weeks, whereas, in the current study the duration of treatment was beyond six months, in fact more than a year in many of them. This could explain the difference in impairment of orgasm between the two studies.

This is a single contact hospital-based study done to test the hypothesis formulated, based on the available literature, and aimed at assessing the frequency of sexual dysfunction involving risperidone, olanzapine, and quetiapine and comparing them. Although a cross-sectional design is not always the best design for such studies, the problem of attrition seen in the prospective studies is not an issue here. Only the clinically stable patients were incorporated with a careful assessment on BPRS, as the patients' account is less reliable during the symptomatic phase. However, full remission is rarely achieved in schizophrenia, especially with respect to negative and cognitive symptoms. The current study is, to some extent, similar in methodology to that of Smith and colleagues.[6] However, the latter has not used BPRS. They have used the Calgary Depression Inventory to rule out depression among patients with schizophrenia, and the UKU side effect rating scale to assess the autonomic side effects. Various questionnaires addressing sexual function have been used in different studies.[2,21–23] The problem with most of them is that the same questionnaire is not replicated in several further studies to enhance its validity. Furthermore, the reliability and validity of data collected by means of questionnaires are jeopardized by intentional nonreporting or over-reporting, incomplete recall, misunderstanding of survey questions, and selective participation. Therefore, the questionnaire is not entirely responsible for the credibility of the data. The current study has used the original SFQ, designed by Smith and colleagues, without any modification. It was based on the evidences that men with schizophrenia were able to answer direct questions regarding concrete aspects of sexual functioning. A sexual partner was not necessary to answer the questions in SFQ. In our patient population, the frequency of sexual dysfunction was much higher than in the original study by Smith and colleagues. This could be attributed to differences in factors such as dose and duration of treatment of the sample population, differing psychosocial environments, inherent biological variation among races, apart from the patient-related errors in answering the questionnaire as mentioned above. This study did not incorporate the determination of biological markers like the serum prolactin level, unlike other studies.[17,18,24,25] Such an estimation could have further strengthened it.

The duration of antipsychotic exposure is an important factor in impaired sexual functioning. In case of risperidone, the literature says that it behaves as a typical antipsychotic in doses of more than 6 mg/day. However, it might have the same effects even in lower doses when given for several years. Thus both dose and duration may have equally important roles. Any difference in sexual dysfunction due to higher and lower doses of each drug has not been compared in this study, as this was not the focus of this study. However, such a comparison may yield useful implications. This is another limitation of the present study. In the study by Bobes and co-researchers, although quetiapine was found to have lesser sexual dysfunction compared to risperidone and haloperidol, it was used for a shorter duration (12 weeks) and the author cites this as a limitation of his study.[7] In the present study though the duration of treatment among the three drugs was evenly distributed, there was a significant difference in the dose of study drugs. The chlorpromazine equivalent dose of quetiapine was significantly greater than the other study drugs, as mentioned earlier. This might be a significant factor in this study, as higher sexual dysfunction was observed with quetiapine compared to the earlier studies. This also indicates that quetiapine may be needed in relatively higher doses to bring about clinical stability in schizophrenia.

## CONCLUSION

Thus the results of this study allow no concrete conclusion to be made as to which atypical antipsychotic is significantly safer than the other as far as the sexual side effects are concerned. However, in future, such a research with similar methodology should involve the comparison of drugs with the chlorpromazine equivalent doses evenly matched. An attempt should also be made to compare individual drugs at higher versus lower doses. A study of prolactin levels is also a useful complimentary procedure. Also higher the sample size, better the inference. With all such modifications being implemented in future research, we could probably arrive at a reliable conclusion.
